# Benchmark datasets and software for developing and testing methods for large-scale multiple sequence alignment and phylogenetic inference

**DOI:** 10.1371/currents.RRN1195

**Published:** 2010-11-18

**Authors:** C. Randal Linder, Rahul Suri, Kevin Liu, Tandy Warnow

**Affiliations:** ^*^Integrative Biology, University of Texas; ^†^University of Texas; ^‡^The University of Texas and ^§^Microsoft Research New England, and The Department of Computer Science, University of Texas at Austin

## Abstract

We have assembled a collection of web pages that contain benchmark datasets and software tools to enable the evaluation of the accuracy and scalability of computational methods for estimating evolutionary relationships. They provide a resource to the scientific community for development of new alignment and tree inference methods on very difficult datasets. The datasets are intended to help address three problems: multiple sequence alignment, phylogeny estimation given aligned sequences, and supertree estimation. Datasets from our work include empirical datasets with carefully curated alignments suitable for testing alignment and phylogenetic methods for large-scale systematics studies. Links to other empirical datasets, lacking curated alignments, are also provided. We also include simulated datasets with properties typical of large-scale systematics studies, including high rates of substitutions and indels, and we include the true alignment and tree for each simulated dataset. Finally, we provide links to software tools for generating simulated datasets, and for evaluating the accuracy of alignments and trees estimated on these datasets. We welcome contributions to the benchmark datasets from other researchers.

One of the principal goals of the National Science Foundation's Assembling the Tree of Life (AToL) initiative is “[a]ssembly of a framework phylogeny, or Tree of Life, for all major lineages of life.”[1]  Much of that effort has focused on accumulating and analyzing data for the major taxonomic groups.  However, because of the scale of the problems (numbers of species and amount of sequence information), the initiative has also required development of methods for sequence alignment, phylogenetic inference and supertree estimation that can handle hundreds, thousands or even tens of thousands of sequences.  In the last decade, many new methods have been developed to address these challenging computational problems, including RAxML [2], GARLI [3], POY [4], SATé [5], and MrBayes [6].  However, evaluations of the efficacy of these methods for large-scale alignment and tree estimation--required for highly accurate estimations of the Tree of Life--have lagged behind method development. 

To facilitate testing of large-scale alignment and phylogeny estimation methods, we have assembled a collection of web pages of (1) benchmark datasets and (2) software appropriate for creating new simulated benchmark datasets (http :// www . cs . utexas . edu / users / phylo / datasets /).  Because these datasets have been assembled with an eye to their usefulness for Tree of Life-scale projects, only datasets that have large numbers of taxa and/or present other difficulties for phylogenetic reconstruction and alignment (e.g., high rates of substitution and insertions and deletions) are included.   The datasets we provide range in numbers of taxa from a few hundred to more than 300,000 sequences.  The datasets are broken down into sets most appropriate for three types of phylogenetic problems: phylogenetic estimation given aligned sequences,  supertree estimation, and multiple sequence alignment.  Some datasets are appropriate for more than one type of problem and therefore are referenced more than once.  Reference information and links are provided for all published datasets.   

## Benchmarks for phylogenetic estimation 

The benchmark datasets for phylogenetic estimation are both empirical and simulated.  They have been used in large-scale systematics studies, and so present challenges for maximum likelihood, maximum parsimony and Bayesian estimation.  A subset of the empirical datasets (Table 1) include curated alignments and reference trees (generated using RAxML version 7.0.4 [2]).  Reference trees have been assessed by bootstrapping, with edges having less than 75% support contracted.  The remaining empirical datasets lack curated alignments and reference trees, but are appropriate for assessing the ability of alignment and phylogenetic software to operate on large and/or difficult datasets.  They can also be used to compare how well algorithms solve particular optimality criteria, e.g., maximum parsimony or maximum likelihood.  The empirical datasets include both nucleic acid and amino acid sequence data.  

**Table:** 

**Table 1**. Empirical datasets and their properties.						
**Dataset^a^**	**Gene**	**Taxonomic Range**	**Number of Taxa**	**Number of Characters^b^**	**Percentage Indels**	**Average Gap Length**
16S.B.ALL	16S rRNA	Bacteria	27,643	6,857	80.0	4.9
16S.T	16S rRNA	The three domains of life plus mitochondria and chloroplasts	7,350	11,856	87.4	12.1
16S.3	16S rRNA	The three domains of life	6,323	8,716	82.1	9.4
16S.M.aa_ag^c^	16S rRNA	Mitochondria	1,028	4,907	82.6	22.0
16S.M	16S rRNA	Mitochondria	901	4,722	78.1	17.2
23S.M	23S rRNA	Mitochondria	278	10,738	83.7	31.9
23S.M.aa_ag^c^	23S rRNA	Mitochondria	263	10,305	83.5	34.2
23S.E.aa_ag^c^	23S rRNA	Eukaryotes nuclear	144	8,619	61.1	13.5
23S.E	23S rRNA	Eukaryotes	117	9,079	59.7	12.6
^a^Unless otherwise noted, all datasets in this table are taken from Cannone et al. [7]. Curated alignments were produced by Cannone et al. using covariation and secondary structure. The reference trees reported on our web site were generated using RAxML version 7.0.4. Complete run parameters and program commands are listed on the web site. ^b^The number of columns in the aligned dataset. ^c^ [8]						

Simulated datasets (Table 2) were taken from three sources and include both amino acid and nucleic acid sequences of widely varying numbers of sequences, rates of substitution and sizes and rates of indels.  

**Table:** 

**Table 2**. Simulated sequence datasets and some of their properties.					
** Dataset**	**Source**	**Data Type^a^**	**Number of Taxa**	**Number of Characters^b^**	**Software^c^**
FastTree	Price et al. [9] Price et al. [10]	AA NA	250; 1,250; 5,000 78,132	N/A	Rose [11]
SATé	Liu et al. [5]	NA	100; 500; 1,000	1,000	SeqGen [12] Rose [11]
RNASim	kim.bio.upenn.edu/software/csd.shtml	NA (SSU rRNA)	128; 256; 512; 1,024; 2,048; 4,096; 8,192; 16,384; 1,000,000	1,542	RNASim [13]
^a^AA = amino acid; NA = nucleic acid ^b^The number of characters in the root sequence ^c^The software used to generate the datasets					

## Benchmarks for multiple sequence alignment 

Most of the benchmark datasets for multiple sequence alignment are the same as those for phylogenetic estimation.  Both empirical and simulated datasets are provided. Taken as a whole, these datasets have properties that are typical both of markers currently used in large-scale phylogeny estimation and of markers that have evolved under high rates of indels, and are thus extremely difficult to align. 

In addition to the simulated datasets for phylogeny estimation, a simulated amino acid dataset is included [14], which has sequences that were generated using Rose [11] and ranging from 20 to 100 taxa.  Several additional empirical amino acid datasets are also included (BAliBASE [15], OXBench [16], PREFAB [17], SABmark [18]).  

The empirical benchmark datasets for testing multiple sequence alignment include datasets with highly reliable curated sequence alignments that have been carefully validated by the community.  The gold standard for this sort of dataset is Robin Gutell's Comparative RNA Website (CRW) [19].  The curated alignments provided in CRW are based upon secondary structural information, which is particularly helpful where the mature rRNA is double stranded due to sequence complementarity.  

## Benchmarks for supertree methods 

Finally, we provide benchmarks for testing supertree methods.  As with the other benchmark collections, we provide both empirical (Table 3) and simulated [20] supertree datasets, and include datasets with different properties (such as the number of source trees, and the taxon sampling strategies used to produce the source trees).  

**Table:** 

**Table 3**. Empirical supertree datasets.			
**Dataset**	** ** **Taxonomic Range**	** ** **Total Taxa**	** ** **Number of Source Trees**
** ** McMahon and Sanderson [21]	Comprehensive papilionoid legumes	2,228	39
Cardillo et al. [22]	Marsupials	267	158
Beck et al. [23]	Placental mammals	116	726
Kennedy and Page [24]	Seabirds	121	7
Wojciechowski et al. [25]	Temperate herbaceous papilionoid legumes	558	19

## Software for generating datasets 

The collection of simulation software is useful for three aspects of producing simulated datasets: generating simulated phylogenetic trees (e.g., r8s [26] and Mesquite [27]); evolving sequences on phylogenetic trees (particularly tools that evolve sequences with both substitution and indel events) and tools for post-processing model trees in order to deviate from the model assumptions (such as the molecular clock).

## Conclusions

We hope that having these benchmark datasets in a single location will facilitate research on large-scale phylogenetic methods and enhance the reproducibility of work based upon the datasets. We also hope that posting simulation tools that are capable of generating large-scale phylogenetic datasets will promote the generation of new benchmark datasets for use by the community.

We conceive of these pages as an evolving resource for the community and welcome input that would improve or expand them.  We invite readers to contact us if they wish to contribute benchmark datasets or software to this resource or if they know of additional datasets or software that should be added to the pages.  We will be happy to work with the laboratories providing them, and will either store them locally or provide links to their sites. 

**Acknowledgements**

The authors thank the editors and two anonymous reviewers for their very helpful suggestions on the manuscript.  Correspondence should be sent to CRL.  The research was supported by the US National Science Foundation DEB 0733029, and by Microsoft Research through support to TW.

**Funding Information**

The research was supported by the US National Science Foundation DEB 0733029, and by Microsoft Research through support to TW.

**Competing Interests**

The authors have declared that no competing interests exist.
